# A coadapted community-based participatory group programme for parents/carers of children with complex neurodisability (Encompass-2): a pilot and feasibility study protocol

**DOI:** 10.1186/s40814-025-01619-3

**Published:** 2025-05-03

**Authors:** Kirsten Prest, Angela Harden, Kirsten Barnicot, Michelle Heys

**Affiliations:** 1 School of Health and Psychological Sciences, City, University of London, London, UK; 2 Specialist Children’s and Young People’s Services, East London NHS Foundation Trust, London, UK; 3 UCL Great Ormond Street Institute of Child Health, London, UK

**Keywords:** Neurodevelopment, Neurodisability, Cerebral palsy, Community-based programmes, Child(ren), Caregivers, Peer support, Co-design, Feasibility, Acceptability

## Abstract

**Background:**

Parents/carers of children with complex neurodisability continue to lack appropriate family-centred care. “Encompass” is a community-based group programme that was co-adapted from “Baby Ubuntu” in Uganda. It is an example of a “decolonised healthcare innovation” as it is a low-cost solution from a low-income country for use in a resource-constrained UK National Health Service (NHS).

**Methods and analysis:**

We will conduct a mixed-methods pilot feasibility study to determine the feasibility and acceptability of delivering and evaluating “Encompass” with parents/carers of children under 5 years with complex neurodisability in the UK. We aim to recruit 20 parents/carers of children from two NHS trusts in England serving urban areas where there is high social deprivation and ethnic diversity. Recruited parents/carers will attend the 10-modular, participatory group programme over a 6-month period. Groups will be facilitated by a trained allied health professional and an “expert parent” with lived experience. The primary outcomes of interest are the feasibility of delivering and evaluating the programme (recruitment, retention rates, acceptability as perceived by the parents/carers, facilitators and wider key NHS partners), intervention fidelity and participant adherence. Results will be collectively assessed against traffic light criteria. Pre-, post- and follow-up data collection questionnaires will include the Family Empowerment Scale (FES), the Power Ladder Question, the Parent Patient Activation Measure (P-PAM), Warwick-Edinburgh Mental Wellbeing Scale (WEMWBS), EuroQoL-5D-5-level (EQ-5D-5L) and parent/carer greatest needs and goals questionnaire. Post-intervention semi-structured interviews will be conducted with parents/carers, facilitators and key NHS partners.

**Discussion:**

A community-based participatory group programme is a potentially affordable and sustainable way for the NHS to provide family-centred support. The programme aims to improve outcomes for parents/carers of children with complex neurodisability. Example outcomes include knowledge, skills, confidence, wellbeing and quality of life. The programme also provides opportunities for peer support and aims to empower parents/carers in navigating community health systems.

**Registration:**

The protocol is registered on clinical trials.gov (ID: NCT06310681).

**Ethical approval:**

Health Research Authority ref. 23/EM/0213.

**Supplementary Information:**

The online version contains supplementary material available at 10.1186/s40814-025-01619-3.

## Background

The term neurodisability is commonly used in the United Kingdom (UK) to refer to a group of children or young people with long-term health conditions due to a neurological cause that results in functional impairments in their daily life [1]. An example of a complex neurodisability is cerebral palsy (CP), which describes a group of disorders that are caused by damage to the developing brain not only affecting movement and posture but also providing a range of possible developmental challenges in the form of musculoskeletal, cognitive, sensory, behaviour and communication difficulties [2].

Parents/carers raising children with complex neurodisability face numerous challenges, particularly during key transition periods including the time just after diagnosis. There is a deluge of evidence that explores the physical, psychological and practical challenges that they face [3–7] along with well-established evidence that holistic, family-centred care is best practice [8]. There remains an implementation gap as families continue to describe challenges in accessing appropriate family-centred care. This was demonstrated in a preliminary study (Encompass 1) undertaken in one of the sites where this pilot and feasibility study will take place [9]. In Encompass 1, parents/carers described the challenges in communicating with healthcare professionals, finding the appropriate medical jargon-free information that they require about their child’s diagnosis and services available, and the lack of joined-up holistic care in the community [9].

The study will take place in two urban boroughs in England where there is high social deprivation and ethnic diversity. Those residing in these areas experience significantly higher prevalence of poor mental and physical health, as well as higher service use compared to the UK [10, 11]. One of the boroughs has the lowest proportion of first-language English speakers compared to all local authorities across England and Wales [12]. Both boroughs have higher rates of children living in poverty (44% and 48% of children) compared to the average rate of 33% of children in the city [13]. Health literacy is another example of a health inequality that parents/carers experience in these areas, which can result in poorer health outcomes for their child [14]. The prevalence for low health literacy nationally is 41%, while the prevalence for the study sites is 67% and 58% [15].

To address aspects of this implementation gap in providing appropriate family-centred care, there are a number of parent/carer group programmes being developed and tested in high-income countries globally which include “Healthy Parent Carers” [16, 17], “ENVISAGE (ENabling VISions and Growing Expectations)” [18, 19], “Healthy Mothers, Healthy Families” [20] and “Parenting Acceptance and Commitment Therapy (PACT)” [21]. These programmes target families who have children with a variety of developmental disabilities, and each has slightly differing aims and objectives. Another example of a parent/carer group programme for families of children with complex neurodisability is the “Ubuntu” model. Unlike the other programmes described, “Ubuntu” was initially developed for children with cerebral palsy and those with complex motor disorders, rather than being agnostic about diagnosis. The “Ubuntu” programme includes the children themselves within the group which is another key difference compared to the other programmes. It is therefore more targeted to meet the specific needs of families who have children with complex neurodisability, which is why it was chosen for this study.

Ubuntu (previously known as “Getting to know Cerebral Palsy”) is a community-based participatory caregiver group programme that has been tested in resource-limited settings such as Uganda, Ghana and Bangladesh and has been implemented in low- and middle-income countries globally. Evaluations of this programme demonstrated improvements in parental confidence and self-efficacy, as well as improved quality of life (QoL) for children with cerebral palsy and their families [22–24]. The modular, facilitated, participatory programme is comprehensive in that it aims to promote inclusion and participation for the child with a neurodevelopmental disability (such as CP) in the community, to maximise the child’s health and development, to empower caregivers through information sharing and peer support, to address stigma and to promote the human rights of children with disabilities. The Encompass 1 study also explored the theoretical acceptability and feasibility of adapting and implementing the “Ubuntu” programme in the UK. Most participants described the need for a programme like “Ubuntu”, reporting that it would have been welcome when their children were younger particularly in the period just after diagnosis. The results will be published elsewhere.

“Baby Ubuntu”, an adapted version of “Ubuntu” for babies and young children with developmental disabilities, has been co-adapted using the ADAPT framework [25] to form the “Encompass” programme. The adaptation process and outcome will be published separately. This adaptation and implementation of “Baby Ubuntu” in a high-income context such as the UK is an example of a “decolonised healthcare innovation” as it brings a frugal innovation developed in a resource-constrained setting to a high-income setting such as the UK [26]. There is encouragement to test frugal innovations in the UK National Health Service (NHS) in particular, as it faces a workforce crisis and high constraints in resources [27]. Decolonisation in global health challenges dominant discourses about health created by those in power [28]. The adaptation of “Baby Ubuntu” does this by acknowledging the valuable innovations and knowledge created in low- and middle-income countries regarding optimal ways to holistically support parents of children with disabilities. It also challenges traditional “top-down” approaches common in high-income country healthcare systems by introducing a community-owned, participatory, cost-conscious and peer-led programme. This is part of a recent shift emphasising the importance of community participation in health interventions in high- and middle-income countries [29]. The next step is therefore to pilot the intervention in two settings in the UK NHS using a participatory approach [30], to assess the feasibility of delivering and evaluating the programme to inform a protocol for a larger-scale evaluation. The cost of running the programme will be explored too.

This protocol describes Encompass-2 — a non-randomised pilot and feasibility study.

### Objectives

#### Primary objective

To determine the feasibility and acceptability of delivering the co-adapted community-based group programme (“Encompass”) with parents/carers of children under 5 years with complex neurodisability in urban areas with high levels of social deprivation and ethnic diversity in England.

- To assess the feasibility of intervention delivery (i.e. recruitment, retention rates, acceptability as perceived by the parents/carers, facilitators and wider key NHS partners) to inform the next stage in the development of a large-scale evaluation of “Encompass”
- To assess intervention fidelity and participant adherence

#### Secondary objective

To determine the feasibility of carrying out an evaluation of the “Encompass” programme, which assesses health-related outcomes of parents/carers of children with complex neurodisability, as well as cost-effectiveness.

- To assess the proportion of participants who complete the post-intervention and follow-up assessments
- To explore how participants experience the research visits and the acceptability of the study questionnaires/ assessments
- To assess the appropriateness, acceptability and completeness of the outcome measures
- To assess the means and standard deviations of participants’ scores on the measures at baseline and post-intervention and to determine what pre-post effect sizes are obtained to inform sample size calculations for a future larger-scale evaluation
- To record the cost of the “Encompass” programme delivery and pilot feasibility study phases

## Methods

This is a mixed-methods pilot and feasibility study that aims to determine the feasibility and acceptability of delivering and evaluating the “Encompass” programme to two groups of parents/carers of children with complex neurodisability (< 5 years) recruited from two NHS trusts in England serving urban areas with high levels of social deprivation and ethnic diversity. The protocol has followed reporting guidelines for pilot and feasibility trials from Thabane and Lancaster [31], who suggested utilising elements and adapting the SPIRIT (Standard Protocol Items: Recommendations for Interventional Trials) guidance [32] and the CONSORT extension to pilot trials [33]. Checklists for the SPIRIT and the CONSORT extension may be found in Additional files 1 and 2.

### Theories and frameworks

Four theoretical frameworks will guide the methodology of the study, namely: the Medical Research Council (MRC) framework for developing and evaluating complex interventions [34], the ADAPT guidance [25], the Theoretical Domains Framework [35] and the Context Compass Framework [36].

The pilot/feasibility study is embedded within the MRC framework for developing and evaluating complex interventions [34]. The “Encompass” intervention is a complex intervention due to the multiple components, expertise required to deliver it and its interactions with the wider systems. These systems include health, social and educational services, the local community and global perceptions around disability including stigma. This stage of the study will fall within the feasibility and develop intervention phases of the MRC framework while considering the core elements of context, developing and refining the programme theory, engaging interested parties and identifying key uncertainties. The programme theory has been developed in the form of a logic model using realist methodology and will be published elsewhere along with the process of adaptation.

Clearly defined prompts and questions in the ADAPT guidance aim to deepen the researcher’s understanding of adapting interventions for new contexts [25]. It is recommended to be used alongside intervention development guidance, in this case the MRC framework. The ADAPT guidance recommends evaluating feasibility through recruitment and retention rates, which have been included in the research questions.

Topic guides for qualitative data collection and the subsequent analysis will be guided by the Theoretical Domains Framework (TDF) for individual-level determinants that influence the implementation of the “Encompass” programme (e.g. participants and facilitators) [35]. When the study has a health equity lens, a greater emphasis on context has been recommended [37]. In order to appropriately determine both the setting and system contextual factors that influence implementation, the Context Compass framework will be utilised [36]. Topic guides may include questions about the fit or readiness of the setting to receive the “Encompass” programme, which will be particularly important to discuss with key NHS partners.

To pilot-test and assess the feasibility of the intervention, elements from the CONSORT extension to pilot and feasibility studies will be drawn upon [33], for example it is recommended that the primary aim of a pilot study relates to feasibility of proceeding to a definitive trial and that there be formal progression criteria to decide whether to proceed or not. The CONSORT extension will be used in combination with guidelines created by Lancaster and Thabane [38] which support the preparation and reporting of non-randomised pilot and feasibility studies by providing advice for adapting the CONSORT extension for non-randomised studies. The collection and analysis of qualitative data in this phase will be considered through the lens of O’Cathain et al.’s guidance on maximising the impact of qualitative research in feasibility studies for randomised controlled trials [39]. Feasibility studies aim to gain a deeper understanding of how the intervention works and to facilitate ongoing adaptation and preparation for larger-scale evaluations, and this is where qualitative data may be particularly valuable.

### Study setting

This is a multicentre study with “Encompass” implemented with parents and carers recruited from two NHS trusts in England serving urban areas with high levels of social deprivation and ethnic diversity. Local collaborators will be identified from each site.

The collaborators will be responsible for the local administration of the project by directly identifying potential participants from the clinical database and initiating the approach by providing the study information sheet to parents. They will also be involved in the recruitment of facilitators who will deliver the intervention.

We will access and recruit families in receipt of health and social care services at each participating NHS Trust, as well as professionals employed by both NHS trusts.

### Patient and public involvement

A patient and public involvement (PPI) group was formed during the initial phases of the study setup. The group consists of four mothers who live in the study areas, and all have a child with a complex neurodisability. The group met approximately every 3–4 months in the first year to build rapport, to discuss the study logistics and review any participant-facing documents and to co-adapt the manual and delivery plan for “Encompass”.

### Participants and recruitment

The study population are as follows:

1. Parents/carers of children with a diagnosed and disclosed neurodisability (for example cerebral palsy), who reside in the study areas
2. Children of the above parents/carers
3. Facilitators responsible for the delivery of the coadapted parent/carer group programme. The facilitator team will include a healthcare professional (likely physiotherapist or occupational therapist) and an “expert parent” with lived experience.
4. Key NHS partners involved in the delivery and commissioning of health and care services, for example clinical managers, service leads or commissioners.

**Fig. 1:**
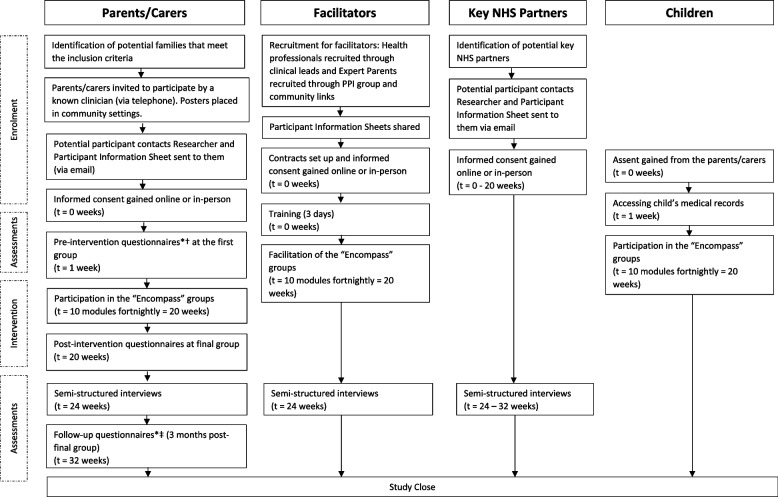
Flow of participants. *Family Empowerment Scale (FES), Power Ladder Question (PLQ), Parent Patient Activation Measure (P-PAM), Warwick-Edinburgh Mental Wellbeing Scale (WEMWBS), EuroQoL-5D-5-level (EQ-5D-5L), †parent/carer greatest needs and goals, ‡intervention satisfaction survey

A SPIRIT diagram presents the planned flow of participants in Fig. 1 and the eligibility criteria in Table 1 below.

**Table 1:** Inclusion and exclusion criteria

	Inclusion	Exclusion
Parents/ carers	Parents and carers who are as follows: • Care for a child (< 5 years at the time of enrolment) with a complex neurodisability^a^ • Have received a diagnosis for their child, which has been disclosed to them, even if this is a diagnosis such as SWAN (Syndrome Without A Name) • Reside in the boroughs of Newham or Tower Hamlets, East London • ≥ 18 years of age	• Have a child with a developmental disability where there are no functional physical impairments as part of their complex needs. For example, children diagnosed with autism spectrum disorder, attention-deficit hyperactivity disorder, and intellectual impairments will be excluded unless they have a functional physical impairment with a neurological cause too • Have a child with a progressive neurological condition such as Duchenne’s Muscular Dystrophy as the family’s needs would be different from those whose child has a life-long condition but is not progressive • Have a child with a structural physical impairment not caused by a neurological event or neurological difficulties. For example, children born with a limb difference or a child with hearing loss • Do not have capacity to consent • Do not meet the inclusion criteria as specified above • There are no exclusions based on language, as interpreting/translating services will be offered
Children	Children of the above parents/carers	The same above exclusion criteria regarding diagnosis apply
Facilitators	1. The health professional who facilitated the “Encompass” groups. The inclusion criteria for this facilitator were as follows: • Therapists or healthcare professionals who work with children with disabilities and who are open to learning with families about their children • Need to be registered with the Health and Care Professionals Council and should ideally have > 5-year post-graduate experience working with a paediatric population • Confident in working with children with complex neurodisability, such as cerebral palsy 2. The expert parent who facilitated the “Encompass” groups. The inclusion criteria for this facilitator were as follows: • Parents/carer of children with complex neurodisability who are identified through other services as potentially being able to facilitate a group as an “expert parent” • Prior experience in training or using participatory approaches — not essential	• Inability to read and speak English • Inability to commit to a 6-month period of work to the best of their knowledge
Key NHS partners	Staff from the NHS that are either involved in the delivery or commissioning of community child healthcare services in Newham or Tower Hamlets Examples include clinical managers, service leads or commissioners	

^a^Complex neurodisability for this study is based on need over a specific diagnosis. Children should have the following: A non-progressive neurological disorder either caused by a congenital brain abnormality or an acquired long-term condition caused by a neurological event (e.g. Hypoxic-Ischemic Encephalopathy or Traumatic Brain Injury), resulting in: A functional physical impairment and Additional difficulties with cognition, hearing and vision communication, emotion and behaviour can form part of the child’s clinical picture, but functional physical difficulties must be present

### Eligibility criteria

#### Recruitment

We will ensure recruitment of parents/carers of children with a known diagnosis of a complex neurodisability, such as cerebral palsy, with wide-ranging insights and experiences including clinical features, demographics (including family structure) and service use history. Potential participants will be approached by a known clinician and recruitment posters placed at NHS sites.

We will make concerted efforts to engage and recruit from those families who historically have been less engaged with clinical services (determined by the children and young people service use history). Participants in the Encompass 1 study were asked their opinions on how to reach more people for the intervention. Parents/carers and healthcare professionals gave suggestions which included the use of posters placed in libraries, general practice (GP) surgeries, schools or community groups. It was suggested that invitations should make it clear that interpretation services will be available.

We do anticipate some recruitment challenges which were explored in the Encompass 1 study. Parents with older children with complex neurodisability report that they would have liked to be invited to a programme such as “Encompass” in the early stages of their journey. However, there was acknowledgement that this can be an overwhelming time, and adding additional appointments to family’s schedules might be a barrier. This study will be important in exploring the feasibility of recruiting parents/carers into the programme, for example through recording reasons for non-participation.

#### Parents/carers of children with complex neurodisability 

The clinical team caring for the children with complex neurodisability will identify eligible cases and gain their consent from parents/carers to be contacted by the research team.

Parents/carers who are interested in taking part will be asked if they agree to being contacted by the research team. They will then email or phone potential participants to explain the study and provide them with a participant information sheet (PIS) and informed consent form. Potential participants will be invited to take time to read and review the study documents and have an opportunity to ask any questions. Informed consent will either be taken in person (via a signed hard copy form) or remotely (via a signed copy form).

Parents/carers who attend the group will have the option of an interpreter if required. Selected parents/carers will take part in semi-structured interviews after the programme, where their travel or data costs will be covered along with interpreter services.

#### Facilitators 

We aim to recruit a healthcare professional (such as a physiotherapist or occupational therapist) with appropriate experience of working with children with complex neurodisability, as well as an “expert parent” with lived experience to facilitate the group programme together.

The healthcare professional role will be advertised in community child health services in East London, and the expert parent role will be advertised through parent/carer form mailing lists as well as online support groups. The PPI group will provide further suggestions on different groups and charities to approach as well as any connections via their children’s schools or activities.

Facilitators will be paid for their time in line with NHS agenda for change pay scales [40] and National Institute for Health and Care Research (NIHR) payment guidance for members of the public considering involvement in research [41].

### Key NHS partners

Key NHS partners in the local health and care system such as clinical managers or commissioners will be identified with the support of the local collaborators.

### Intervention

**Fig. 2:**
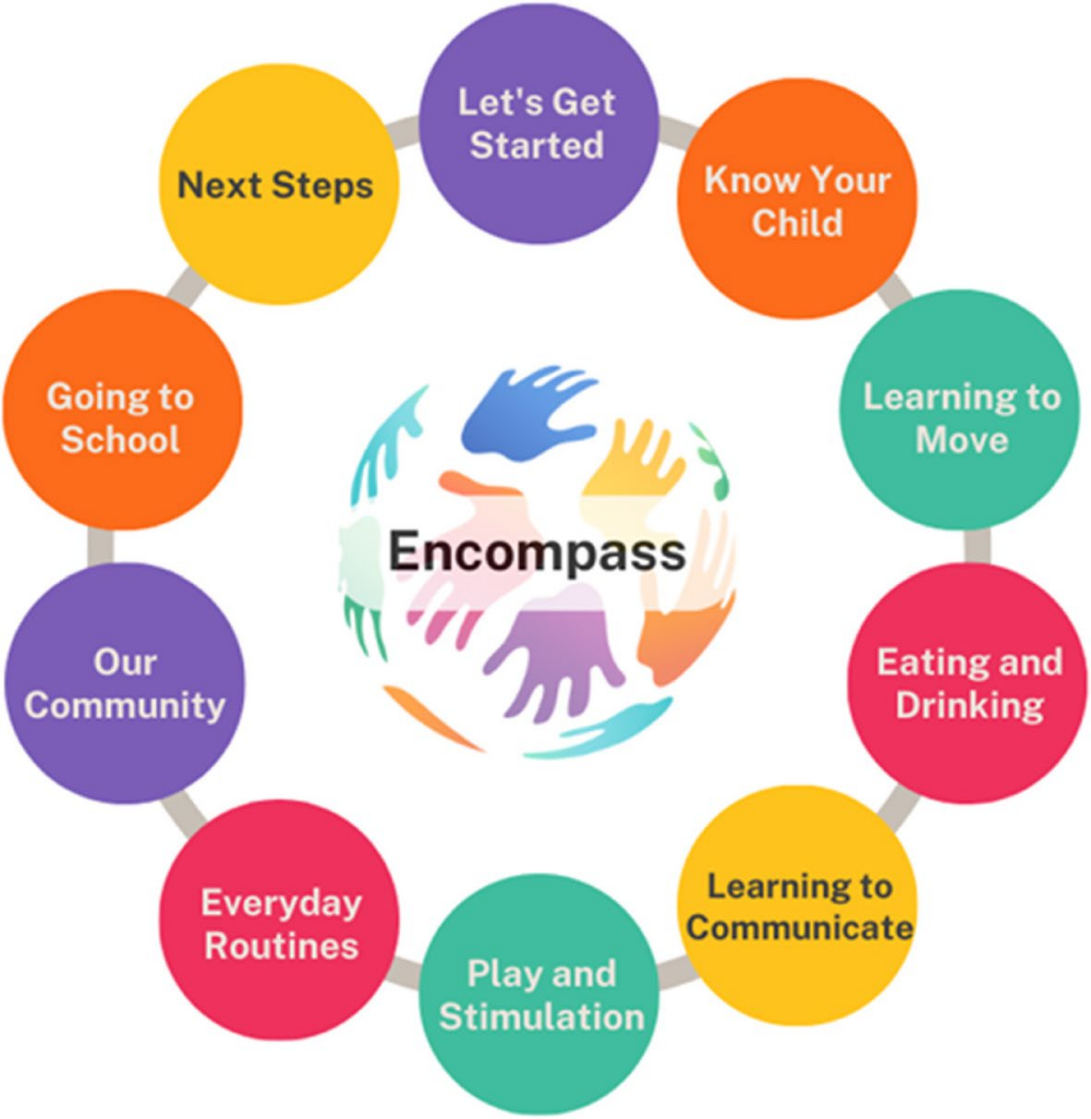
The “Encompass” programme modules

“Encompass” is a 10-module facilitated, group participatory programme for parents/carers of children with complex neurodisability under the age of 5 years (Fig. 2).

“Encompass” aims to run 10 group modules which will follow the topics in Fig. 2. All sessions will be run in person at a community venue such as the local library meeting room. The aim is to run modules fortnightly. Groups will be facilitated by two people: a healthcare professional and an “expert parent”. Facilitators will undergo 3–5 days of training with a master facilitator of the original “Baby Ubuntu” programme.

Families will be invited to the groups from the point at which their child is diagnosed and up to the age of 5 years at the time of enrolment. Siblings will be invited to the “Play and Stimulation” sessions, and any other carers or family/community members will be invited to the “Our Community” session. Handouts will be provided at the end of each group. Group rules/contract will be discussed developed together at the initial group with the assistance of the facilitators.

### Outcomes

#### Primary outcomes

Feasibility, acceptability and process outcome measures will be used. Quantitative data will be collected to assess the traffic light criteria (Table 2). The green light signifies that criteria for progression have been met and a larger evaluation could proceed, the amber light suggests certain amendments need to be made and the red light indicates that the criteria for progression have not been met and researchers should not continue to a trial [42]. The criteria for progression will be collected using the following:

- Study record: An enrolment log will record all eligible participants, total enrolled, reasons for non-participation, number followed up on the individual-level outcomes and the date on how many people responded to adverts/invitations. Reasons will be recorded for those who are eligible but decline to enrol.
- Process measurements: Fidelity checklist and qualitative observations of the intervention groups, group register and post-intervention survey from parent/carer participants. The survey has been adapted from previous evaluations of Ubuntu interventions and is scored on a Likert scale for satisfaction with the content, organisation and facilitators.
- Qualitative data: Semi-structured interviews conducted with parent/carer participants, facilitators and wider key NHS partners (e.g. NHS service managers and commissioners) to explore the acceptability of the intervention.

**Table 2:** Traffic light criteria for the larger scale evaluation

	**Green light**	**Amber light**	**Red light**
**Recruitment — percentage of eligible participants who consent to take part**	35% who are eligible consent to participate	15–34% who are eligible consent to participate	Less than 15% who are eligible consent to participate
**Group attendance**	More than 80% of parents/carers attend the group for 6 + sessions	30–79% of parents/carers attend the group for 6 + sessions	Fewer than 30% attend the group for 6 + sessions
**Follow-up response rate — self-complete outcomes questionnaire(s)**	70% or greater response to follow up	50–69% response to follow-up	Less than 50% response to follow-up
**Fidelity — delivery on items described in the fidelity checklist**	70% or greater score on the checklist	50–69% score on the checklist	Less than 50% score on the checklist

#### Secondary outcomes

The proposed outcomes and evaluation methods for the larger-scale evaluation will include the following:

- Proposed individual level outcomes:
  i. Family empowerment
  ii. Parent patient activation and health literacy
  iii. Parent/carer wellbeing
  iv. Parent/carer quality of life
  v. Parents/carers perceived greatest needs (baseline only) and goals for the intervention (baseline and endpoint)
  vi. Review of goals achieved (endpoint only)
- Economic outcome
  i. Childhood cost calculator (C3): A costing tool for education and early childhood development [43]

The above outcome measures proposed for the larger evaluation will be assessed in the response, completion rates and acceptability during data collection. Table 3 summarises the outcome measures and data collection timing.

**Table 3:** Outcome measure and data collection timing for proposed outcomes and evaluation methods for the larger-scale evaluation

Outcome	Outcome measures	Timing			
		Baseline	End of each “Encompass” group	Post-intervention	3-month follow-up
Proposed individual-level outcomes	Family Empowerment Scale (FES)	X		X	X
	Empowerment ladder	X		X	X
	Parent Patient Activation Measure (P-PAM)	X		X	X
	Warwick-Edinburgh Mental Wellbeing Scale (WEMWBS)	X		X	X
	EuroQoL-5D-5-level (EQ-5D-5L)	X		X	X
	Parent/carer greatest needs and goals	X		X	
Economic outcomes	Childhood Cost Calculator (C3)				X
Process outcomes	Fidelity checklist		X		
	Direct observation		X		
	Group register		X		
	Intervention satisfaction survey			X	
	Qualitative semi-structured interviews			X	

### Family empowerment

The Family Empowerment Scale (FES) [44] is a validated 34-item instrument that measures parents’ sense of empowerment across three areas: family, service use and community. It has been used in the ENVISAGE parent workshops [18] and aligns with the goals of the “Encompass” groups which are to empower families to understand their child’s diagnosis and how to navigate health systems. A limitation of this tool is that it has mostly been used with a white, United States (US) population [45].

The Power Ladder Question (PLQ) assesses participants’ perceived sense of power and influence over their life. The survey asks: “Please imagine a nine-step ladder, where on the bottom, the first step, stand people who are completely without rights, and on the highest step, the ninth, stand those who have a lot of power. On which step are you?” [46]. It has been used with diverse groups and allows the participant to choose the domains of power that they value and interpret the question openly [47].

### Healthy literacy and patient activation

Parent patient activation relates to the knowledge, skills, confidence and persistence to manage a child’s health care, particularly those with developmental disorders or disabilities [48–50]. The Parent-Patient Activation Measure (P-PAM) is a validated 13-item tool that measures two factors: “confidence and knowledge” and “action and perseverance” [51]. It has been used in a variety of diverse settings, including low income, non-English-speaking parents [52].

### Parent/carer wellbeing

The Warwick-Edinburgh Mental Wellbeing Scale (WEMWBS) has been widely used to assess wellbeing across a diverse range of public health interventions, populations and settings [53, 54]. The 14-item scale WEMWBS will be used with five response categories that are summed up to provide a single score [55].

### Parent/carer quality of life

The EuroQoL 5-dimension questionnaire is a validated and widely used tool that measures generic quality of life. It has one question for each of the five dimensions (mobility, self-care, usual activities, pain/discomfort and anxiety/depression) with five response options [56].

### Parent/carer needs and goals

A questionnaire has been adapted from previous evaluations of Ubuntu interventions [57]. It has two questions and asks parents/carers what their three biggest issues are that they face in everyday life and what their two main goals are for attending the group.

### Qualitative data

Qualitative data collection in the form of semi-structured interviews will take place with 4–6 purposefully sampled parent/carer participants per group to reflect a variety of perspectives (e.g. low/high attendance, severity of their child’s difficulties, age or gender), along with both facilitators (*n* = 2) and key NHS partners (*n* = 2–4). As qualitative research is iterative, the semi-structured interview questions will be open-ended, and the direction cannot be fully anticipated. However, the topic guides indicate the broad topics that will be discussed with each participant. The guides were developed based on the TDF and the Context Compass Framework, along with input from the PPI and advisory groups.

Semi-structured interviews with parents/carers will explore their satisfaction with and perceived impact of the intervention, their level of participation in the community, confidence levels, experiences of discrimination and the impact of their child’s disability. They will also be asked their opinions about the data collection tools and outcome measures that were used.

The topic guides for the facilitator interviews were developed based on previous evaluations of the Ubuntu interventions, the Theoretical Domains Framework and the Context Compass Framework. These interviews aim to explore the facilitators’ experiences of how the intervention ran, perceived impacts of the intervention and how it may be integrated into existing services.

Semi-structured interviews with key NHS partners from the local health and care system will explore the acceptability of incorporating the “Encompass” intervention into current services and pathways, potential facilitators and barriers, theoretical feasibility and cost-effectiveness. They will also be asked their views on outcomes that should be measured in a larger-scale evaluation.

### Background demographic data

Basic background demographic data will be collected at the first groups from the parents/carers. This will be combined with background data accessed from the children’s medical records about their diagnosis and interactions with different health services.

#### Data management and access

Data will be managed and overseen by City, University of London. Semi-structured interviews will be audio-recorded, and either transcribed verbatim by a professional transcriber or transcribed through Teams if it took place online. Transcripts will be pseudo-anonymised so that no individual or organisation can be identified from the data.

All digital recordings, anonymised transcripts and other person-identifying research data will be stored in password-protected files on secure servers at City, University of London. Only the immediate research team will have access to these files.

Digital recordings will be destroyed at the end of the study. Anonymised research data will be held on City, University of London servers for 10 years.

A password-protected database of participant contact details will be stored separately from the anonymised research data. This will be held on City, University of London secure servers, and only the research team will have access to these files. This database will be erased at the end of the study.

### Sample size

The sample size of the study was generated based on a variety of literature and methods. Simple confidence interval calculations were used for the feasibility estimates research questions and qualitative justifications for the acceptability research questions.

As the objectives of the feasibility study relate to estimating a rate (i.e. the proportion of people) of those who completed follow-up questionnaires and attended the groups, it is suggested that confidence intervals may be calculated by relating the proposed sample size to the width of the confidence interval for the rate, using the following equation with *P* being the proportion one expects to see and *n* the intended sample size [58]:

$$1.96 \times \surd (P \times (1-P)/n)$$

The standard error of a proportion depends on the value of the proportion itself, reaching its largest value when the proportion equals 0.50 [58]. The table below displays the width of confidence intervals across reference sample sizes for two values of proportion (Table 4):

- An estimation of the follow-up response rate questionnaires being 70%
- As estimation of group attendance (attending > 6 out of 10 modules) of 80%

**Table 4:** Width of confidence intervals across reference sample sizes for two values of proportion (completion of follow-up questionnaires and group attendance)

*n*	Group configuration		*P*	CI_95_
12	Two groups of six each	Follow-up	0.70	0.26
		Attendance	0.80	0.23
16	Two groups of eight each	Follow-up	0.70	0.22
		Attendance	0.80	0.20
20	Two groups of 10 each or 3 groups of 6 or 7 each	Follow-up	0.70	0.20
		Attendance	0.80	0.18
24	Three groups of eight each	Follow-up	0.70	0.18
		Attendance	0.80	0.16
30	Three groups of 10 each	Follow-up	0.70	0.16
		Attendance	0.80	0.14

For a follow-up response rate of 70% in the feasibility study of 12 participants, we can be 95% confident that this estimate is accurate within + / − 26%. When the sample size is increased to 20 participants, the error in estimation is reduced to 20 percentage points. If increased to 24 participants, this is only slightly reduced to + / − 18%. An increase to 30 participants results in a marginal reduction to + / − 16%.

The above calculations relate to the feasibility rate estimates and suggest that a sample of 20 may be adequate.

As this is a mixed-methods study, qualitative data will be collected around the acceptability of delivering and receiving the intervention. Information power [59] has been proposed as a tool to guide sample size in qualitative research. It suggests that a sample with greater information power requires a lower *n* and vice versa. The model proposes that certain considerations will require either the least amount or a larger number of participants. The aim of the qualitative data collection is narrow (exploring the acceptability of delivering and receiving the intervention), and the researcher (K.P.) is confident in her abilities to conduct interviews with strong dialogue due to her background as an occupational therapist and recent experience in conducting a similar qualitative study. The theoretical background is strong as there have been multiple studies globally that have explored the same topic, albeit in different contexts. Participants for the qualitative data collection are required to have highly specific characteristics that have not been previously described, for example residing in a high-income country with ethnic and linguistic diversity. These characteristics of aim, specificity, dialogue and theory enhance information power resulting in fewer participants required. Based on the above, a sample size consisting of the facilitators (*n* = 2), parents/carer (*n* = 8 to 12) and key NHS partners involved in local health and care system commissioning and management (*n* = 2 to 4) may be sufficient for the qualitative study.

### Data analysis

Recruitment rates, completion rates for baseline and follow-up outcomes and attendance rates will be assessed against the traffic light criteria.

Quantitative data, such as the Family Empowerment Scale and satisfaction survey, will be descriptively summarised using mean and standard deviation for continuous variables and number with percentages for categorical variables.

Qualitative data will be audio-recorded and transcribed, and NVivo software will be used to manage and organise the data. Data will be analysed thematically [60] both deductively and inductively. Analysis will be guided by the TDF and the Context Compass Framework described previously. By developing the topic guides using these frameworks, it ensures that key contributors to feasibility are explored. Thus, data that will be coded and analysed deductively within the TDF and Context Compass frameworks to develop qualitative themes that likely to map to the framework domains. However, it is also likely that other themes may arise from the data that do not map to the domains of these frameworks (inductive analysis).

During data synthesis, the research team will aim to look for congruence and incongruence between qualitative and quantitative findings, as well as attempting to use qualitative data to clarify quantitative findings.

### Ethics

Ethical approval has been obtained from the Health Research Authority (ref. 23/EM/0213). Key considerations include the researchers having a clear understanding of the informed consent procedures, with it being emphasised that declining to participate or withdrawing from the study will not affect a child’s healthcare in any way. Appropriate procedures are in place for safeguarding if any participant discloses inappropriate clinical practice or indicates that they or their family may be at risk of harm at any time over the course of this research. Participant confidentiality and data protection have been considered throughout. All personal data will be collected, stored and processed in accordance with the Data Protection Act 2018 and General Data Protection Regulation. Participants will be informed of their rights to confidentiality and the rights of others; however, limits to confidentiality will also be explained; for example, in the case where a parent or child may be at risk of harm, the research team may need to notify external parties to protect the safety of parents/children.

There is a risk of parents/carers feeling emotional distress in the group setting. This phase of the study includes parents/carers with young children who are either newly diagnosed or in the process of being diagnosed with a complex neurodisability. At this stage, parents/carers may be experiencing difficulties with their mental health [4] and feelings of denial, anxiety and worries about the future, as was seen in the Encompass 1 study. Parents/carers with newly diagnosed children with CP often find comfort in a group setting; however, it can at times be too emotionally difficult to see others within the group, particularly those with more severe physical difficulties [61]. Facilitators of the groups will be experienced physiotherapists or occupational therapists, as well as other parents with lived experience, who will receive training about supporting parents/carers’ needs around the time of diagnosis. Signposting will be provided for further psychological support if required.

### Dissemination

On completion of the study, the data will be analysed and prepared for a final study report in the form of a PhD thesis. This will be stored in the City, University of London library for general access. The student researcher (K.P.) will prepare work for publication, in collaboration with the research team, during the different project phases. The main findings from this study will be published in open-access peer-reviewed journals, presented at conferences, and through public engagement. Members of the PPI group will be invited to contribute to dissemination activities.

The findings will be made available on the “Encompass” study website and the Ubuntu-Hub website. Module materials will be made available to download on one of these sites.

## Supplementary Information

Supplementary Material 1.

Supplementary Material 2.

Supplementary Material 3.

Supplementary Material 4.

## Data Availability

Not applicable.

## Abbreviations

CP: Cerebral palsy
CYP: Children and young people/person
EQ-5D-5L: EuroQoL 5-dimension scale
FES: Family Empowerment Scale
GP: General practitioner
HCP: Healthcare professionals
HRA: Health Research Authority
MRC: Medical Research Council
PIS: Participant Information Sheet
PLQ: Power Ladder Question
P-PAM: Parent-Patient Activation Measure
PPI: Patient and Public Involvement
SPIRIT: Standard Protocol Items: Recommendations for Interventional Trials
QoL: Quality of life
REC: Research Ethics Committee
TDF: Theoretical Domains Framework
UK: United Kingdom
US: United States
WEMWBS: Warwick-Edinburgh Mental Wellbeing Scale
