# Supplementary material for: A coadapted community-based participatory group programme for parents/carers of children with complex neurodisability (Encompass-2): a pilot and feasibility study protocol
Source: Pilot Feasibility Stud. 2025 May 3;11:59. doi: 10.1186/s40814-025-01619-3 (PMC12048988; doi:10.1186/s40814-025-01619-3)
Supplement: Supplementary file 3 — Supplementary Material 3. [file 40814_2025_1619_MOESM3_ESM.docx]

**Parent/Carer Facilitators**

We are inviting you to take part in a research study taking place at the Specialist Children’s and Young People’s Service (SCYPS) in the East London Foundation Trust and the Children’s Therapy Services in Tower Hamlets in the Barts Health NHS Trust. Before you decide whether to take part, it is important that you know why we are doing the study and what is involved. Please read the following information carefully.

This research study is part of a project called ENCOMPASS, which is a group of studies that aim to explore community-based group programmes that support families of children with complex neurodisability. This ENCOMPASS project is part of a PhD fellowship funded by the HARP PhD programme and City, University of London will be the Data Controller.

**What is the purpose of the study?**

We have co-adapted a community-based group programme for parents/carers of children with complex neurodisability that was initially developed in countries such as Bangladesh, Ghana and Uganda. We now plan to test the programme in East London, UK. This is the first time this programme has been adapted for a country like the UK and so we are testing whether it can be delivered here, and how we can collect data to see if it works. If you would like to read about previous adaptations of the programme, visit the Ubuntu Hub <https://www.ubuntu-hub.org/>.

We would like to see whether the programme is acceptable to parents/carers and facilitators in Newham and Tower Hamlets, as there are many different ethnicities and languages spoken. We would also like to see whether it is practical to be delivered within the NHS.

**Why have I been chosen?**

We are recruiting parent carers to facilitate the “Encompass” groups, alongside a healthcare professional. The only requirement is that you have a child with a complex neurodisability and a desire to support other parents/carers who may be earlier along in their journey. We are also looking for the following broad criteria:

- The ability to work together as a team with a healthcare professional such as a physiotherapist or occupational therapist.
- Be able to commit to a six-month period (excluding school holidays) for one morning (3.5 hours) every two weeks.
- Be familiar with the local context of Newham or Tower Hamlets.
- Some prior experience in training or some prior experience in using participatory approaches for training or in community-based projects would be beneficial but not essential

**What would taking part involve?**

“Encompass” aims to run ten group modules which will follow the topics in Figure 1. It will aim to run all sessions in person at a community venue such as the local library meeting room. Modules will aim to run fortnightly. You will undergo 3-5 days of training alongside the other facilitators with a master facilitator who has undergone the master training and who is usually a trained therapist. The training will prepare you with facilitation skills and will help you become familiar with the content of the programme manual. You will have the support of the Principal Investigator (KP) who
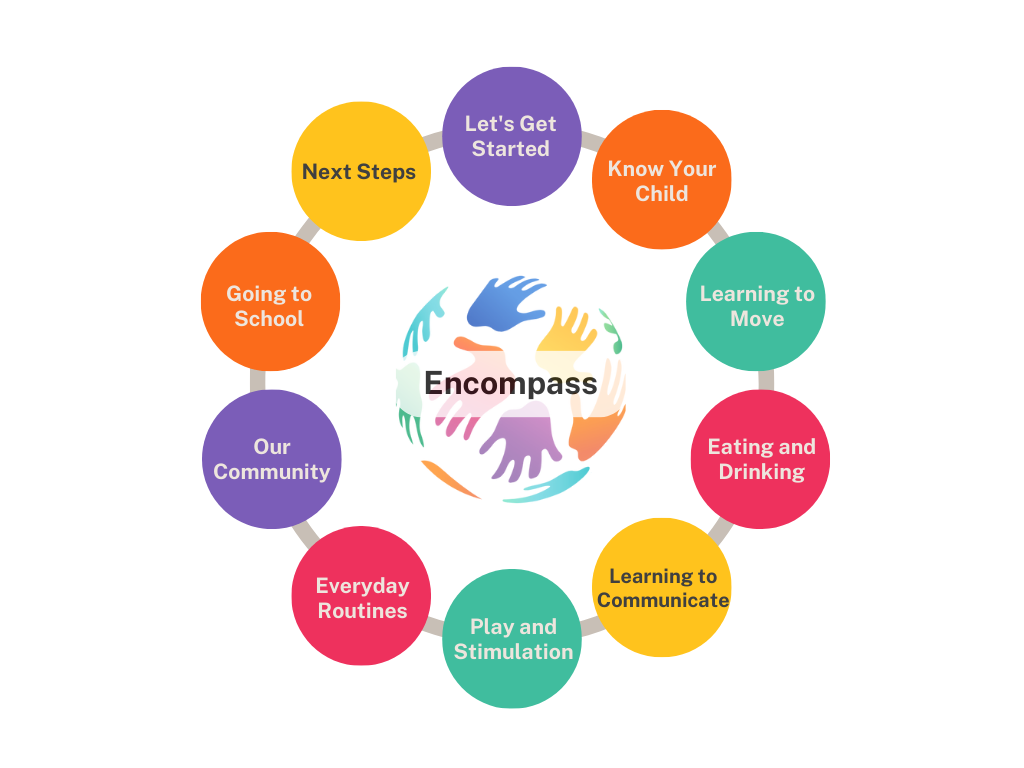
is a master trainer

**Figure 1: The “Encompass” programme modules**

After the groups have been completed, We will invite you, the facilitators, who have carried out the “Encompass” groups to take part in an interview to explore the experiences of delivering the programme and whether you think it is feasible and acceptable to be delivered in an East London NHS setting.

**Will you record the interview?**

Yes, we will ask for your consent to audio and/or video record the interview. Please refer to page 4 to see how we will use this information.

**How much time will it take?**

The training will run over three full days or five half days. The ten group sessions will be run fortnightly over a six-month period to account for school holidays. Each session will be two hours, however we would require you to be there for half the day to account for setting up, packing away and debrief/feedback.

The interview will be approximately 1 hour.

**How many people are involved in this research?**

We hope to involve around 20 parents/carers and four trained facilitators (two health professionals and two parents/carers with lived experience). Two facilitators will be based in Tower Hamlets, and two in Newham. You will be able to provide cover for each other if needed.

**Do I have to take part in this research?**

No. It is up to you to decide whether you would like to take part.

**Will you reimburse my costs?**

Yes, we will reimburse your time for the facilitation of the groups in line with NIHR payment guidance, and your travel costs for the interview. If you participate in a remote interview (e.g. on the phone or skype) we will give you a voucher to cover data costs if needed (i.e. top up mobile phone voucher) instead of travel expenses.

**What are the possible benefits of taking part?**

You will help with the development of a group programme that supports parents/carers of children with complex neurodisability. You will be helping parents and carers who are earlier on in their journey of having a child with a complex neurodisability. You could provide support and information that you may have wanted or needed at that time in your life. You will also develop valuable skills in facilitating groups and working together with a healthcare professional.

**What are the possible disadvantages of taking part?**

Facilitating the Encompass groups will expose you to parents/carers with newly diagnosed children with complex neurodisability who are still coming to terms with the diagnosis and beginning their journey. You will have appropriate training for when parents/carers express distress and there will be time to feedback and debrief after each group. If you are finding it difficult to manage, more in-depth supervision can be organised. Your participation is voluntary, and you may withdraw from the study if needed.

Whilst the interview is unlikely to result in distress, if this was to occur, we would be able to take a break from the interview. Also please remember your participation is voluntary and you are able to withdraw at any stage.

**How will we use information about you?**

If you choose to enrol in this study, we will ask you for your contact details. This includes: your name, address, mobile number and email address. This will be kept confidential. Only the Principal Investigator will have access to your contact details. They will destroy this information at the end of the study.

Any audio and video recordings made will be kept securely. Only the research team and an external transcriber will have access to the recordings. The recordings will be destroyed at the end of the study.

A typed-up record of your answers (transcript) will be kept for a longer period. This will be stored anonymously. This means that your name, and any other identifying information, will not be linked to what you have said.

This anonymised information will be shared among members of the research team. We will also use this anonymous information in research reports and publications. In the future, it may also be shared with other researchers at other institutions.

Direct quotes from interviews may be used in research journal publications, however any identifying information (e.g. names) will be removed from the quotes.

We will destroy all copies of the anonymous study data after 10 years.

In the unlikely event that you find yourself unable to make decisions for yourself while participating in the study, we will have to stop your involvement. However, any information we have collected from you up to that point will still be kept securely and used as part of the research, without revealing your identity in order to maintain your privacy.

**Limits to Confidentiality**

If you tell us anything in the interview or discussion that makes the Principal Investigator worry that either you or others are at risk of harm, the Principal Investigator may have to inform their supervisors. If this happens, the Principal Investigator will discuss this with you, before they do this.

**What are your choices about how your information is used?**

You can stop being part of the study at any time by letting the Principal Investigator know. You do not have to give a reason.

If you decide to withdraw, the Principal Investigator will ask what you would like us to do with your data. You can request to have your personal data (e.g. any records of your name and contact details) erased at any time. However, we will keep the research data that you have already given us (e.g. your responses in interviews and group discussions). This information will not be linked to your name or contact details. Your data will have a code (number) instead.

**Where can you find out more about how your information is used?**

You can find out more about how we use your information:

- By asking one of the research team (see details at the end)
- By visiting City University’s website about how they handle data <https://www.city.ac.uk/about/governance/policies/data-protection-policy>

**What will happen to the findings of this study?**

The findings of this study will be written up as part of a thesis for a Professional Doctorate in Health Services Research. We will also publish the findings of this research in reports, scientific journals and our website. When we report what you have told us we will not use your name or any other personal identifying information about you. Instead, we will replace your name with a code (number). In this way, the information you give us will be anonymised.

We will also hold some events in Newham and Tower Hamlets to share our findings with the wider community. We will invite you to attend and participate in these events.

**Who is organising and funding this study?**

The research is being organised by Kirsten Prest (Chief Investigator) who is a HARP PhD Fellow at City, University of London and an Occupational Therapist at the East London NHS Foundation Trust (ELFT). The study is funded by the HARP PhD Programme.

**How have patients and the public been involved in this study?**

We asked four parents of children with complex neurodisability from Newham and Tower Hamlets to give their views on all the research documents (e.g. consent forms, questionnaires and information sheets). We also asked for their advice and guidance on recruitment procedures. We have also worked with these parents through the adaptation of the programme, to gain their views and insights.

**Who has reviewed this study?**

This study has been independently peer-reviewed and has received regulatory approvals. The study has received HRA approval from the Health Research Authority (HRA) and Favourable Ethical Opinion from the Research Ethics Committee (REC).

**What if there is a problem?**

If you have any problems, concerns, or questions about this study, you should ask to speak to a member of the research team (see page 6). If you remain unhappy and wish to complain formally, you can do this through City’s complaints procedure. To complain about the study, you can phone 020 7040 3040. You can then ask to speak to the Secretary to Senate Research Ethics Committee and inform them that the name of the project is *ENCOMPASS: Pilot testing of a co-adapted group programme for parents/carers of children with complex neurodisability.*

You can also write to the Secretary at:

Annah Whyton
Research Integrity Manager

City, University of London, Northampton Square, London, EC1V 0HB

Email: [Annah.whytton@city.ac.uk](mailto:Annah.whytton@city.ac.uk)

**Further information and contact details**

If you would like to talk to someone about the study, please contact:

Chief Investigator: Kirsten Prest

Email: [Kirsten.prest@city.ac.uk](mailto:Kirsten.prest@city.ac.uk)

Local Principal Investigator:

Email:

PhD research supervisor at City, University of London: Professor Angela Harden

Email: [angela.harden@city.ac.uk](mailto:angela.harden@city.ac.uk)

PhD supervisor at University College London and Consultant Paediatrician at East London NHS Foundation Trust: Professor Michelle Heys

[m.heys@ucl.ac.uk](mailto:m.heys@ucl.ac.uk)

Thank you for taking the time to read this information sheet.
