# Supplementary material for: A coadapted community-based participatory group programme for parents/carers of children with complex neurodisability (Encompass-2): a pilot and feasibility study protocol
Source: Pilot Feasibility Stud. 2025 May 3;11:59. doi: 10.1186/s40814-025-01619-3 (PMC12048988; doi:10.1186/s40814-025-01619-3)
Supplement: Supplementary file 4 — Supplementary Material 4. [file 40814_2025_1619_MOESM4_ESM.docx]

**Parents/Carers**

**Please read the following 13 statements carefully and initial the boxes before signing. Please note that it is not mandatory to consent to items 9- 12 and you can still take part in the study without consenting to these.**

|  |  | Please initial | |
| --- | --- | --- | --- |
| 1. | I confirm that I have read and understood the participant information sheet for the above-named study. I have had had the opportunity to consider this information and to ask questions and I am satisfied with the answers. |  | |
| 2. | I understand that my participation is voluntary (my choice). I can choose not to participate or to withdraw at any stage without giving any reason, without my medical care or legal rights being affected. |  | |
| 3. | I understand that data collected during the study may be looked at by individuals from regulatory authorities or from the NHS Trust, where it is relevant to my taking part in this research. I give permission for these individuals to have access to my data. |  | |
| 4. | I understand that the information collected about me will be used to support other research in the future and may be shared anonymously with other researchers. |  | |
| 5. | I understand that everything that I say will be treated as confidential, unless there is a concern that my family is at risk of harm. |  | |
| 6. | (If appropriate) I give permission for [Name: ] to be present and to act as my interpreter. |  | |
| 7. | (If appropriate) I agree for my conversations during the interview to be audio recorded. These will be used to understand my experiences of the intervention and provide valuable information for the researchers. |  | |
| 8. | I understand that what I say in the interview may be included in reports and journal articles as quotes but that these will be anonymous and any details about myself or my child will be removed. |  | |
| The following items are optional. Please circle ‘Yes’ or ‘No’ in answer to questions 9-12. | | | |
| 9. | I give permission for photographs to be taken of me during the groups for the purpose of sharing the findings of the research with others at the end of the study (e.g. conferences, local workshops or newsletters). | Yes | No |
| 10. | I give permission for photographs to be taken of my child during the groups for the purpose of sharing the findings of the research with others at the end of the study (e.g. conferences, local workshops or newsletters). | Yes | No |
| 11. | I give permission for members of the research team to access the medical records of my child. | Yes | No |
| 12. | I would like to receive information about the outcome of the study or be invited to future events where the results of the study are shared. For this reason, I give permission for my contact details to be kept after the study has ended. | Yes | No |
|  |  | Please initial | |
| 13. | I agree to take part in the above study. |  | |

_______________ ____________________________ _____________

Name of Participant Signature Date

____________________ ____________________________ _____________

Name of Researcher Signature Date

**File Note**

Original copy to be retained by the research team and one copy to be provided to the participant.
